# Medical treatment of osteoporosis in osteoporotic vertebral fractures - Results from the prospective EOFTT multicenter study

**DOI:** 10.1016/j.bas.2026.106127

**Published:** 2026-06-22

**Authors:** Philipp Schenk, Jessy Wiedemann, Klaus J. Schnake, Ulrich J.A. Spiegl, Sebastian Katscher, Martin Bäumlein, Volker Zimmermann, Gregor Schmeiser, Michael A. Scherer, Michael Müller, Katja Liepold, Kai Sprengel, Simon Schramm, Christopher Baron, Georg Osterhoff, Alexander Franck, Max J. Scheyerer, Bernhard Ullrich, Falko Schwarz, Working Group Osteoporotic Fractures of the Spine Section of the German Society of Orthopaedics and Trauma

**Affiliations:** a Innovationhub for Musculoskeletal Surgery Halle, BG Klinikum Bergmannstrost Halle gGmbH, Halle, Germany; b Department of Trauma, Reconstructive and Hand Surgery, Städtisches Klinikum Dresden Friedrichstadt, Germany; c Center for Spinal and Scoliosis Surgery, Malteser Waldkrankenhaus St. Marien, Erlangen, Germany; d Department of Orthopedics and Traumatology, Paracelsus Private Medical University Nuremberg, Nuremberg, Germany; e Clinic for Trauma Surgery and Orthopaedics, Munich Harlaching, Sanatoriumsplatz 2, München, 81545, Germany; f Interdisciplinary Center for Spine and Neurotrauma, Sana Klinikum Borna, Borna, Germany; g Center for Orthopaedics and Trauma Surgery, University Hospital Giessen and Marburg GmbH, Marburg, Germany; h Department of Trauma and Orthopedic Surgery, Klinikum Traunstein, Traunstein, Germany; i Department of Spine Surgery, Schoen-Klinik Hamburg-Eilbek, Hamburg, Germany; j Lehrkörper Medizinische Fakultät der Technischen Universität München (Med. Fak. TUM), Munich, Germany; k Department of Orthopedic and Trauma Surgery, University Medical Center Schleswig-Holstein, Campus Kiel, Germany; l Department of Spine Surgery, Thuringia Clinic “Georgius Agricola” Saalfeld, Teaching Hospital of the University of Jena, Saalfeld, Germany; m Hirslanden Clinic St. Anna, Praxis medOT, Faculty of Health Sciences and Medicine, University of Lucerne, Lucerne, Switzerland; n Department of Trauma Surgery, University Hospital Erlangen, Erlangen, Germany; o Spinal Cord Unit, BG Trauma Center, Tubingen, Germany; p Department of Trauma Surgery and Orthopaedics, BG Klinikum Unfallkrankenhaus Berlin, Germany; q Centrum für Muskuloskelettale Chirurgie, Charité-Universitätsmedizin Berlin, Berlin, Germany; r Department of Orthopaedics and Trauma Surgery, Sana Klinikum Coburg, Germany / Ortho Sport GbR, Coburg, Germany; s Department of Orthopedic and Traumatology, University Hospital Duesseldorf, Duesseldorf, Germany; t Department of Trauma and Reconstructive Surgery BG Klinikum Bergmannstrost Halle gGmbH, Germany; u Department of Trauma and Reconstructive Surgery University Medicine Halle, Halle, Germany; v Department of Neurosurgery, Jena University Hospital - Friedrich Schiller University, Jena, Germany

**Keywords:** Osteoporosis, Vertebral fracture, Osteoporotic therapy, Gender differences, Secondary prevention

## Abstract

**Introduction:**

Osteoporotic medication (oTh) is essential for secondary prevention but is often not prescribed after vertebral fractures.

**Research question:**

The aim of this analysis was to assess the status of oTh in inpatients with osteoporotic thoracolumbar fractures and to analyze possible supply gaps.

**Materials and methods:**

Data were collected as part of the multicenter, prospective EOFTT (Evaluation of the Osteoporotic Fracture Classification, Treatment Score and Therapy Recommendations) study. A total of 518 patients with osteoporotic thoracolumbar fractures in 17 clinics were included. The presence, type, and changes over time of oTh were recorded and evaluated using the Cochrane Q test.

**Results:**

Hospitalisation increased oTh with 36% at admission significantly in women to 83%, and in men to 71% (p < 0.001). At admission women received more frequently oTh than men (p = 0.017). At the follow-up after 7 ± 5 months, there was no significant differences between the sexes (p = 0.330). However, the proportion in both groups decreased significantly to 41% (women) and 34% (men), respectively (p < 0.001). In-hospital treatment led to a significant increase in the initiation of anti-osteoporotic medication, with calcium and vitamin D supplementation rising from 13% at admission to 35% at discharge (p < 0.001). However, a substantial and significant decline was observed during follow-up (18%, p < 0.001).

**Discussion and conclusion:**

In the inpatient setting, there is a clear improvement in oTh, which is particularly pronounced in female patients. However, the significant decline in therapy rates at follow-up highlights deficits in long-term care. Despite existing recommendations, evidence-based agents such as bisphosphonates or osteoanabolic are still rarely used. This indicates a relevant gap in care in secondary prevention and underscores the need for structured, post-hospitalisation concepts for sustainable osteoporosis therapy.

## Highlights

• Deficits in the prescription of osteoporotic therapy to patients with osteoporotic fractures.
• Significant increase in medication administration during hospitalisation.
• Shortcoming of prescriptions in the care of male patients.
• Supply gap in the administration of osteoporotic drug combinations.
• Significant decline in medication administration in outpatient care.

## Abbreviations

DGOU: German Society for Orthopaedics and Trauma
DVO: German Society for Osteology
EOFTT Study: Evaluation of the Osteoporotic Fracture Classification, Treatment Score and Therapy Recommendations Study
OF: Osteoporotic fracture
oTh: Osteoporotic therapy

## 1 Introduction

It is well known that the population is aging, particularly in industrialized countries. For this reason, there is also a continuing increase in the prevalence of age-related diseases such as osteoporosis (Hadji et al., 2024). Fragile bones lead to a high number of osteoporotic vertebral fractures every year. In Germany, the number is approximately 100,000–130,000 fractures per year (Lang et al., 2023).

Patients who have suffered a vertebral fracture have up to a 4 times higher risk of sustaining a subsequent fracture (Kanis et al., 2004). Some studies suggest that 15-20% of those affected suffer a subsequent fracture. Exercise therapy and osteoporotic therapy are options to prevent fractures.

Depending on the study, medication can reduce the risk of subsequent fractures in 40-70% of cases (Black et al., 2007; Cummings et al., 1998). Despite this evidence, there are still significant deficits in clinical practice: In a survey of members of the German Spine Society, less than half of the participants reported prescribing combination therapy in accordance with guidelines; a significant proportion did not initiate any osteoporotic therapy, the initiation of therapy in treatment-naive patients was often delayed, and only about one-third stated that they regularly applied the DVO guideline (Bekele et al., 2025).

In many cases, however, medication is not administered. Possible reasons for this could be that the treating physicians are unsure about how to use the medication correctly or that they do not consider initiating drug therapy.

The present analysis aims to investigate the use or administration of osteoporotic therapy in patients with vertebral fractures. In addition, the study focuses on identifying possible gender-specific differences and gaps in care in these patients.

## 2 Material and methods

The present analysis is based on data from the EOFTT study (Clinical Evaluation of the Osteoporotic Fracture Treatment Score (OF-Score): Results of the Evaluation of the Osteoporotic Fracture Classification, Treatment Score and Therapy Recommendations), a prospective, multicenter observational study conducted at 17 spine centers in German-speaking countries (Germany and Switzerland) (Ullrich et al., 2023). The EOFTT study was designed to clinically evaluate the Osteoporotic Fracture (OF) classification, the OF-Score for treatment recommendation in patients with osteoporotic thoracolumbar vertebral fractures (Schnake et al., 2018). Patients were treated either conservatively or surgically at the discretion of the treating physician, in accordance with contemporary recommendations of the Spine Section of the German Society for Orthopaedics and Trauma (DGOU) for osteoporotic vertebral fractures (Blattert et al., 2018). Clinical assessments were performed at three predefined time points: hospital admission, discharge, and follow-up. If more than one follow-up examination was available, data from the latest follow-up were used for analysis. The EOFTT study was approved by the local institutional ethics committees of all participating centers, and written informed consent was obtained from all patients.

In addition to treatment modality (surgical vs. conservative treatment), data collection included age and sex, the presence of trauma as the underlying fracture mechanism, fracture morphology according to the OF classification, and the anatomical location of the fractured vertebra (thoracic spine, thoracolumbar junction, or lumbar spine). Fracture diagnosis was based on radiological imaging, including X-ray, computed tomography (CT), or magnetic resonance imaging (MRI).

Osteoporotic therapy (oTh) was assessed using a standardized questionnaire within the EOFTT study protocol at admission, discharge, and follow-up. The oTh was documented both as the general presence or absence of oTh and in a differentiated manner according to specific medication classes. These included calcium supplementation (e.g., calcium carbonate, calcium citrate), vitamin D preparations (e.g., cholecalciferol, calcitriol), hormonal therapies, bisphosphonates (e.g., alendronate, risedronate, zoledronic acid), and antibody-based therapies (e.g., denosumab).

Osteoporosis was defined in accordance with the recommendations of the German Society for Osteology (DVO) and the World Health Organization (WHO) (Kanis, 1994). In patients older than 75 years, osteoporosis was assumed based on age and fracture presentation, and no additional diagnostic confirmation was required. In patients aged 75 years or younger, bone quality was assessed using dual-energy X-ray absorptiometry (DEXA), quantitative computed tomography (qCT), or opportunistic evaluation of bone density based on Hounsfield unit (HU) measurements on computed tomography scans. HU-based assessment was performed according to the methodology described in literature (Ullrich et al., 2022; Buenger et al., 2022; Simion et al., 2024).

Bone quality parameters (DEXA, qCT, HU) were analyzed descriptively for all available measurements. As multiple assessment modalities were available for some patients, individuals could contribute to more than one modality-specific analysis. Therefore, sample sizes differ between modalities and do not represent mutually exclusive patient groups. For each modality, all available measurements were included without prioritization of a single method.

Comparisons to baseline for age, follow-up duration and bone quality were performed using a generalized linear model (GLM). Crosstabulations with Fisher's exact test were used to assess differences between men and women regarding the presence of trauma, fracture morphology, fracture location, radiological diagnostic method, type of treatment (conservative vs. surgical), and the presence of oTh at the observation time points. Ordinal logistic regression was used to assess the association between the presence of oTh and fracture morphology. Changes in oTh or the concurrent use of a combination of oTh over time with respect to sex and treatment were analyzed using Cochran's Q test. Statistical analyses were performed using SPSS version 29, with a significance level of p < 0.05.

## 3 Results

The mean age of the study population was 75 ± 10 years (p = 0.15). The age range was 41 to 94 years in men and 50 to 97 years in women. No significant differences were observed between men and women regarding the presence of trauma, fracture morphology, or fracture location. The mean follow-up duration was 7 ± 5 months, with no significant difference between men and women in follow-up length (p = 0.411). Table 1 provides an overview of the patients’ descriptive characteristics.

**Table 1:** Baseline characteristics and clinical features of the study cohort stratified by sex. Data are presented for the total cohort and separately for men and women. Categorical variables are reported as absolute frequencies and percentages, and age is shown as mean ± standard deviation.

		Total	Men	Woman	p
N		518	128	390	
Age		75 ± 10	74 ± 10	75 ± 9	0.150
Trauma					0.491
	Yes	168 (32%)	36 (28%)	132 (34%)	
	No	338 (65%)	89 (70%)	249 (64%)	
	Unknown	12 (2%)	3 (2%)	9 (2%)	
OF classification					0.728
	OF1	3 (1%)	1 (1%)	2 (1%)	
	OF2	122 (24%)	26 (20%)	96 (25%)	
	OF3	218 (42%)	59 (46%)	159 (41%)	
	OF4	152 (29%)	36 (28%)	116 (30%)	
	OF5	23 (4%)	6 (5%)	17 (4%)	
Region					0.222
	Thoracic	72 (14%)	13 (10%)	59 (15%)	
	Thoraco-lumbal	367 (71%)	91 (71%)	276 (71%)	
	Lumbal	79 (15%)	24 (19%)	55 (14%)	
Bone density assessment					
	DEXA	276 (53%)	65 (51%)	211 (54%)	0.541
	qCT	147 (28%)	45 (35%)	102 (26%)	0.055
	HU	419 (81%)	103 (80%)	316 (81%)	0.897
Therapy					0.590
	Conservative	344 (66%)	88 (69%)	256 (66%)	
	Surgical	174 (34%)	40 (31%)	134 (34%)	
oTh					
	Admission	184 (36%)	33 (26%)	151 (39%)	0.010
	Discharge	405 (78%)	91 (71%)	314 (81%)	0.017
	Follow Up	203 (39%)	44 (34%)	159 (41%)	0.330

**Fig. 1:**
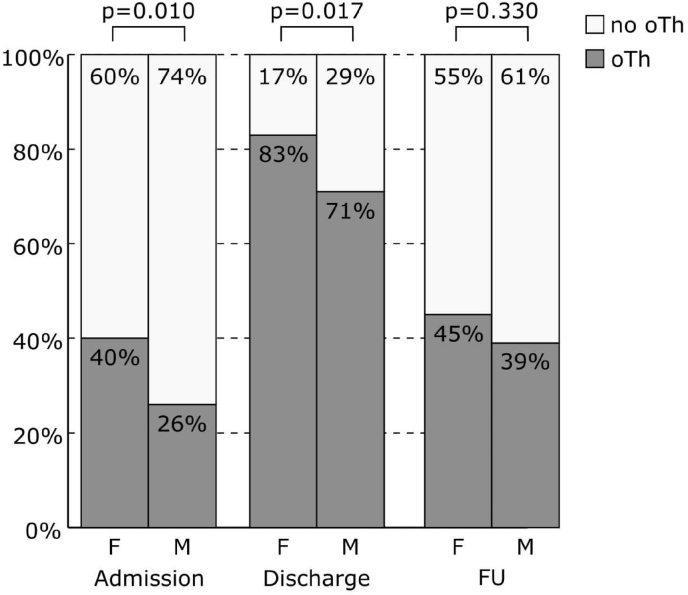
Taking osteoporotic medication (oTh) upon admission, discharge and during follow-up (FU), depending on gender (F- female, M – male).

The proportion of patients receiving oTh increased during the hospital stay (women: from 40% to 83%; men: from 26% to 71%; p < 0.001). The proportion of women receiving oTh was significantly higher than that of men both at admission (p = 0.01) and at discharge (p = 0.017) (Fig. 1). The duration of follow-up did not differ significantly between men and women (7 ± 5 months). In both groups the proportion of patients receiving oTh decreased significantly to 45% in women and 39% in men (p < 0.001, each). In men, oTh was still significantly more frequent at follow-up compared with admission (p = 0.030). In contrast, no significant difference was observed in women between admission and follow-up (p = 0.162).

The ordinal logistic regression showed no significant association (p = 0.257); no relationship was observed between the presence of oTh and fracture severity.

A total of 479 patients (92%) underwent bone quality measures using DEXA, qCT, or HU, or a combination of these methods. Radiological assessment of bone quality most frequently relied on Hounsfield units (HU) derived from CT scans (81%), followed by DEXA (53%), while qCT was used least often (28%). There were no significant differences in the frequency of the applied radiological assessment between sexes (p > 0.055). Bone quality values did not significantly differ between patients with and without oTh at admission (p > 0.273, data given in supplemental material). A combination of at least two of these diagnostic methods was performed in 279 patients (54%), with no sex-related differences observed (p = 0.684). Bone quality did not differ between sexes for any of the three radiological assessment techniques (DEXA p = 0.203, qCT p = 0.352, HU p = 0.552). Bone quality stratified by sex is summarized in Table 2.

**Table 2:** Bone quality values are presented as mean and standard deviation (SD), with minima and maxima given in square brackets, for the entire cohort (total) and separately for men and women with osteoporotic vertebral fractures.

	Total	Men	Women	p
DEXA (N = 276)	−3.2 ± 1.2 [-6.5; 1.7]	−3.1 ± 1.6 [-5.7; 1.7]	−3.3 ± 1.0 [-6.5; 0.5]	0.203
qCT (N = 147)	62 ± 24 [10; 130.4]	65 ± 24 [10; 115]	61 ± 24 [13; 130]	0.352
HU (N = 419)	76 ± 32 [6; 183]	78 ± 32 [12; 172]	76 ± 33 [6; 183]	0.552

With p > 0.065, no significant differences in the frequency of oTh were observed between the two treatment groups (conservative vs. surgical) at any of the three observation time points (admission, discharge, and follow-up, see Table 3). In both treatment groups, the frequency of oTh increased significantly during the period of inpatient treatment and subsequently decreased again at follow-up (p < 0.001). In conservatively treated patients, oTh was more frequently observed at follow-up than at admission (p = 0.029). In surgically treated patients, no significant difference in the frequency of oTh was observed between admission and follow-up (p = 0.232).

**Table 3:** Distribution of osteoporotic therapy in the study cohort by treatment strategy. Data are shown for the total cohort and stratified by surgical and conservative management.

	Total	Surgical	Conservative	p
Admission	184 (36%)	131 (38%)	53 (31%)	0.098
Discharge	405 (79%)	277 (82%)	128 (74%)	0.065
Follow-up	203 (43%)	134 (43%)	69 (43%)	1.000

**Fig. 2:**
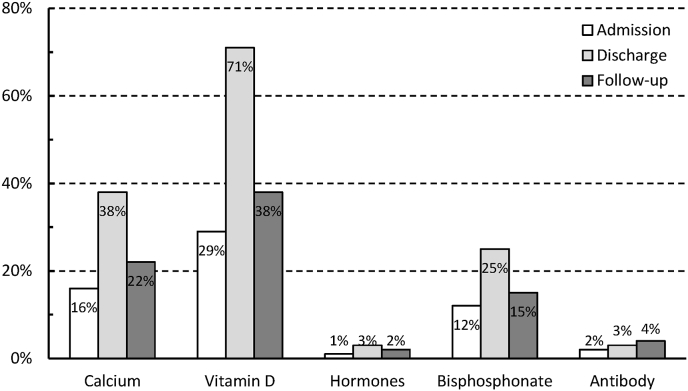
Distribution of osteoporotic therapies at admission, discharge, and Follow-up. Relative frequencies (%) are shown for Calcium, Vitamin D, Hormone therapy, Bisphosphonates, and Antibody-based treatments.

The oTh is observed at three time points and differences between men and women are presented in Fig. 2.

A combination of calcium and vitamin D was observed in 69 patients (13%) at admission. This increased significantly to 179 patients (35%) at discharge (p < 0.001). At follow-up, the number of patients receiving both supplements decreased to 95 (18%, p < 0.001), but remained significantly higher than at admission (p = 0.019). Considering men and women, 8% of men and 15% of women received a combination of calcium and vitamin D at admission (p = 0.036). At discharge, these proportions increased to 30% in men and 36% in women (p < 0.001, each), with no significant difference between the sexes (p = 0.285). At follow-up, no significant differences were observed between men (15%) and women (19%, p = 0.430).

At the time of admission, the combination of calcium, vitamin D, and antibodies was observed in only 3 patients (0.6%, all women, gender p = 1.000). By discharge, in 8 patients this combination was observed (1.5%, 7 women and 1 man, gender p = 0.686). At the follow-up, the combination was found in 9 patients (1.7%, all women, gender p = 0.121). The change in frequency this oTh combination from admission to discharge and from discharge to follow-up was not statistically significant (p = 0.065, p = 0.712).

The combination of calcium, vitamin D, and bisphosphonates was found in 28 patients at the time of admission (5%, 20 women, 8 men, gender p = 0.653). At discharge, this number increased significantly with p < 0.001 to 75 patients (14%, 54 women, 21 men, gender p = 0.472), and then decreased significantly with p < 0.001 to 38 patients at follow-up (7%, 28 women, 10 men, gender p = 0.845).

## 4 Discussion

In this study, we prospectively examined the provision of osteoporotic medications at 17 clinics in Germany and Switzerland. Notably, the number of medications increased significantly during hospitalisation but declined rapidly again in the further course of treatment. In addition, a difference in care between men and women was observed.

It is well known that the number of patients with osteoporosis is increasing, particularly in industrialized countries (Balasubramanian et al., 2019). Very large studies also show an increase in osteoporotic fractures. The prevalence of radiological vertebral fractures increases from 5% to 10% between the ages of 50 and 59 and up to 30% in people over the age of 80 (Ensrud and Schousboe, 2011). In particular, the risk of a subsequent fracture after a previous vertebral fracture is described as 18% (Hadji et al., 2021).

One possible preventive method for reducing secondary fractures is to begin or to intensify osteoporotic therapy (Gregson et al., 2025). This can significantly reduce the number of fractures. Depending on the medication studies report reductions of up to 70% (Black et al., 2007).

Spechbach et al. concluded that early initiation of osteoporotic therapy during hospitalisation leads to better care than delayed outpatient initiation of therapy (Spechbach et al., 2019). We did not investigate this direct correlation in our study, but it was noticeable that there was a significant increase in the initiation of osteoporotic therapy during the inpatient stay, but this fell back to almost the initial level during the last outpatient visit. For this reason, it is important not only to initiate therapy, but also to provide a concept for the physician providing further treatment and, if necessary, to refer the patient to outpatient specialists, as recommended, for example, by Fracture Liaison Services.

There are various recommendations regarding which medications are recommended for osteoporotic therapy. The guidelines of the European Society of Endocrinology as well as the German guidelines on the prevention, diagnosis, and treatment of osteoporosis most frequently mention bisphosphonates (alendronate, risedronate, zoledronic acid, and ibandronate) for the prevention of possible further fractures (Eastell et al., 2019; Stumpf and Schmidmaier, 2024). These are followed by, for example, antibodies such as denosumab. However, due to the high rebound phenomenon, the indication for this should be considered very critically. Teriparatide, abaloparatide, and romosozumab are also recommended for reducing vertebral fractures with a therapy lasting up to 2 years. Selective oestrogen receptor modulators such as raloxifene or bazedoxifene are also among the recommended active substances.

Furthermore, the above-mentioned drugs should be combined with adjuvants such as calcium and vitamin D (Eastell et al., 2019).

In our study, we observed very frequent administration of vitamin D and calcium. In most cases, however, only one of these was administered. Drug combinations, such as the additional administration of an antibody or a bisphosphonate, were very rarely given overall. There is a significant deficit in this area. One possible reason for this is that these drugs have been used very rarely in surgical wards to date, possibly because the treating physician is unsure about their use. One way to increase the administration of these drug combinations would be to develop, introduce and implement concepts, for example as part of a standard operating procedure for patients with osteoporotic fractures during their inpatient stay, in collaboration with an osteologist.

In addition to differences in the use of various osteoporotic drugs, there are also differences in the administration of drugs to different genders. The underprovision of treatment appears to be particularly pronounced in male osteoporosis patients. Kiebzak et al. investigated gender-specific differences in osteoporotic medication after osteoporotic hip fractures as early as 2002 (Kiebzak et al., 2002). In comparison, the data from our study show higher prescription rates at the time of discharge: 71% of men received oTh (vs. 7% in the study by Kiebzak et al.), while the administration for women was 81% (vs. 31%). The difference also persists in the follow-up, but at a higher overall level: in the Kiebzak study, 27% of men and 71% of women received oTh during the course of the study, while in the EOFTT study, 34% of men and 41% of women were treated. Overall, these comparisons indicate an increase in awareness and treatment initiation over the last two decades, with a continuing gender-specific discrepancy.

This discrepancy contrasts with international guideline recommendations. Several guidelines – including recommendations from the National Osteoporosis Foundation, the Endocrine Society and Osteoporosis Canada – advocate osteoporotic treatment for men over 50 years of age following osteoporotic vertebral fractures (D'Amelio and Isaia, 2015). At the same time, the available data show that the medication prescribed consists mainly of basic medication (vitamin D and calcium). However, according to the updated DVO guideline, specific osteoporosis therapy is indicated even in cases of a single low-trauma vertebral fracture of grade 2 or 3 according to Genant or in cases of multiple vertebral fractures (Pfeil and Lange, 2024). Since the OF classification was used in the present study, the proportion of Genant grade ≥2 fractures cannot be determined directly; consequently, the exact quantification of guideline-based underprovision is methodologically limited. Regardless of this, male patients in the present data receive oTh less frequently than female patients across all survey dates. This confirms a gender-specific gap in care also in the context of osteoporotic vertebral fractures and is in analogy to the under-provision described by Kiebzak et al. after hip fracture.

Overall, there seems to be increased clinical awareness of osteoporosis, but implementation of comprehensive care remains inadequate, particularly for male patients.

Several factors could potentially explain this. Firstly, a survey by Chernot et al. points to a discrepancy in primary care: around 80% of general practitioners felt confident in treating osteoporosis, but only around half reported being familiar with the relevant guidelines (Chenot et al., 2007). This could contribute to the fact that oTh initiated in hospital is not consistently continued, adjusted or reinitiated during the course of treatment, thus helping to explain the decline in oTh after discharge. A Europe-wide survey in primary care showed that one third of patients with a documented diagnosis of osteoporosis and an increased risk of fracture still do not receive specific drug therapy. In addition, even after a vertebral fracture had occurred, around 40% of those affected did not receive osteoporotic treatment (McCloskey et al., 2021). Secondly, compliance and adherence are critical factors: Hadji et al. report limited compliance, particularly among men and patients under the age of 60 (Hadji et al., 2016), which could also contribute to lower follow-up rates, even if the therapy was initially prescribed.

Thirdly, it can be assumed that osteoporosis in men continues to be perceived as a less common clinical diagnosis than in women, resulting in less emphasis being placed on diagnosis and treatment, despite clear recommendations for the treatment of men over 50 years of age following osteoporotic vertebral fractures (D'Amelio and Isaia, 2015).

Surgical treatment options are often associated with clearer inpatient responsibilities, structured perioperative procedures and closer interdisciplinary involvement. From this, it could be inferred that osteoporosis is more frequently recognised as a risk factor and that oTh is more likely to be initiated or considered in discharge communication. However, this assumption is not confirmed by the present results: there were no significant differences in the frequency of oTh between conservatively and surgically treated patients at any of the three time points (admission, discharge, follow-up). The decisive factor was rather the time of treatment: in both groups, the frequency of oTh increased significantly during inpatient treatment and decreased again by the follow-up. An interview study at a German maximum care provider shows that osteoporosis is often not systematically recorded in everyday clinical practice because it is not considered to have any immediate consequences in acute situations; in addition, there are obstacles to DXA diagnostics and weaknesses in discharge management and outpatient transition, which means that diagnoses and recommendations are not consistently passed on (Kohalmi et al., 2025).

Our study has several limitations. As this was an observational study, the decision to initiate or withhold anti-osteoporotic therapy was not protocol-driven but based on the treating physician's clinical judgment. Bone quality assessment was one relevant factor, but treatment decisions also considered fracture occurrence, comorbidities, and overall patient condition. Although a significant increase of oTh was observed during hospitalisation, a possible confounding factor could be the study protocol, which reminded doctors to administer osteoporotic therapy. It can be assumed that the actual number of medications administered during hospitalisation is even lower. Furthermore, due to the study protocol, the number of medications administered and the doses of the respective medications were not recorded. In addition, the average follow-up period was relatively short at approximately 7 months, so that a longer-term follow-up would have yielded additional important results regarding the incidence of subsequent fractures. However, our results show a significant decrease in oTh in the first few months, which should raise red flags for health care providers. Finally, we did not include any patients in our study who were only treated on an outpatient care, i.e., patients with minor fractures.

## 5 Conclusions

The number of osteoporotic thoracolumbar fractures is steadily increasing. Adequate osteoporotic medication is crucial to prevent subsequent fractures. Our study reveals significant shortcomings, particularly in the administration of osteoporotic drug combinations and their continuation after discharge. Furthermore, we revealed a shortcoming in the care of male patients. In this regard, the treating physician who diagnoses the fracture, as well as any other physicians involved in the patient's care, should be more aware of the need to initiate and continue a guideline-compliant osteoporotic combination therapy. This could be achieved through close communication with the physicians providing further treatment and by developing an appropriate concept at the beginning of fracture treatment. One way to achieve this is through fracture liaison services.

## Authorship

All authors should have made substantial contributions to all of the following: (1) the conception and design of the study, or acquisition of data, or analysis and interpretation of data, (2) drafting the article or revising it critically for important intellectual content, (3) final approval of the version to be submitted.

## Declaration of competing interests

The authors declare that they have no known competing financial interests or personal relationships that could have appeared to influence the work reported in this paper.
