# Supplementary material for: Medical treatment of osteoporosis in osteoporotic vertebral fractures - Results from the prospective EOFTT multicenter study
Source: Brain Spine. 2026 Jun 22;6:106127. doi: 10.1016/j.bas.2026.106127 (PMC13329376; doi:10.1016/j.bas.2026.106127)
Supplement: Multimedia component 1 [file mmc1.pdf]

Supplementary material

Bone quality modalities of patients with and without oTh

	Total			Men			Women		
	oTh	no oTh	p	oTh	o oTh	p	oTh	no oTh	p
DEXA (N=276)	-3.2±1.2	-3.2±1.1	0.914	-3.1±1.9	-3.0±1.5	0.889	-3.2±1.0	-3.3±1.0	0.823
qCT (N=147)	59±23	64±24	0.294	64±22	65±25	0.927	57±24	63±24	0.273
HU (N=419)	76±34	76±31	0.781	73±27	80±33	0.340	76±36	75±31	0.811

Distribution of osteoporotic therapies at admission, discharge and FU. Data are presented for admission, discharge, and follow-up for the overall cohort and stratified by sex.

		Total	Men	Women	p
Admission	Calcium	85 (16%)	14 (11%)	71 (18%)	0.577
	Vitamin D	151 (29%)	27 (21%)	124 (32%)	0.817
	Hormones	4 (1%)	1 (1%)	3 (1%)	0.562
	Bisphosphonate	60 (12%)	12 (9%)	48 (12%)	0.841
	Antibody	11 (2%)	0 (0%)	11 (3%)	0.222
Discharge	Calcium	198 (38%)	45 (35%)	153 (39%)	0.906
	Vitamin D	370 (71%)	84 (66%)	286 (73%)	0.834
	Hormones	18 (3%)	3 (2%)	15 (4%)	0.774
	Bisphosphonate	130 (25%)	29 (23%)	101 (26%)	1.000
	Antibody	18 (3%)	3 (2%)	15 (4%)	0.774
Follow-Up	Calcium	105 (22%)	24 (21%)	81 (22%)	0.797
	Vitamin D	182 (38%)	37 (32%)	145 (40%)	0.152
	Hormones	9 (2%)	3 (3%)	6 (2%)	0.456
	Bisphosphonate	72 (15%)	20 (17%)	52 (14%)	0.455
	Antibody	21 (4%)	2 (2%)	19 (5%)	0.125
